# Biofunctionalization of Selenium Nanoparticle with *Dictyophora Indusiata* Polysaccharide and Its Antiproliferative Activity through Death-Receptor and Mitochondria-Mediated Apoptotic Pathways

**DOI:** 10.1038/srep18629

**Published:** 2015-12-21

**Authors:** Wenzhen Liao, Zhiqiang Yu, Zehua Lin, Zhuogui Lei, Zhengxiang Ning, Joe M. Regenstein, Jiguo Yang, Jiaoyan Ren

**Affiliations:** 1 College of Light Industry and Food Sciences, South China University of Technology, Guangzhou 510640, Guangdong, China; 2 School of Pharmaceutical Sciences, Guangdong Provincial Key Laboratory of New Drug Screening, Southern Medical University, Guangzhou, Guangdong 510515, China; 3 Department of Food Science, Cornell University, Ithaca, NY, 14853-7201, USA

## Abstract

Bio-functionalized nanoparticles with semiconducting/metallic core encapsulated in a bio- or bio-derived materials are promising for applications in biology and especially in cancer diagnostic and healing. In this report, we report a facile, single-step, first-time synthesis and *in-situ* functionalization strategy for the preparation of monodispersed selenium nanoparticles (SeNPs) functionalized using a novel polysaccharide (DP1) extracted from *Dictyophora indusiata* (a fungus). The DP1 functionalized SeNPs (DP1-SeNPs), where DP1 is attached to the surface via Se-O bond as well as physic-sorption had, an average diameter of 89 nm, and were highly uniform, extremely stable compared to bare SeNPs. Detailed investigation of the biological properties of DP1-SeNP illustrated that they exhibit unprecedented, enhanced, and selective antiproliferative activity through inducing cell apoptosis confirmed by nuclear condensation, DNA cleavage, and accumulation of S phase cell arrest. The mechanism of the induced apoptosis was found to be a combination of the activation of caspases 3, 8, and 9, the Fas-associated death domain protein (FADD), reactive oxygen species (ROS) overproduction, as well as mitochondrial dysfunction. It is envisioned that the reported DP1-SeNPs will offer a new phase space for high-efficiency anticancer treatment with little side effect.

Bio-nanotechnology, the interface of biology and nanotechnology, has made remarkable progress in providing nanophase materials for cancer treatment^1^,^2^,^3^. The unique physicochemical properties, including magnetic, optical, and structural properties which allow them to self-assemble and retain specificity, as well as stability^4^, in combination with efficient penetration, better targeting, and reduced toxicity of nanomaterials made these miniscule materials an outstzanding prospect for next-gen cancer treatment applications. The facile solubility or dispersibility of the medication in aqueous media is a critical challenge which needs urgent attention to implementing nanomaterials-based cancer chemotherapy. Diverse anticancer materials including metals, semiconductors, polymers, and biomolecular materials have been designed and applied for cancer treatment. Among these functional anticancer materials, selenium nanoparticles (SeNPs) based system have gained significant attention as anticancer biomaterials. Especially, selenium (Se) in addition to having excellent photoelectrical and semiconductor properties is an essential micronutrient for human health^4^,^5^ and a key component of selenoenzymes, such as peroxidase, thioredoxin reductase, iodothyronine deiodinases, glutathione peroxidase, etc.^6^,^7^,^8^,^9^,^10^ and can prevent free radicals induced cell damage *in vivo*. It is also established via epidemiological and preclinical studies that seleno-compounds are efficient cancer chemopreventive and chemotherapeutic agents^11^,^12^,^13^. The studies emphasized the exceptional biological activities as well as low toxicity^14^,^15^,^16^,^17^,^18^ of SeNPs and opened-up new horizon for anticancer treatment-based the application of SeNPs. However, before employing SeNPs in therapeutics, lack of stability and toxicity and/or side effects generated by oriented uptake by cellular systems has to be addressed. Moreover, a deep and systematic understanding of the curative effects of SeNPs is still not achieved. The combination of biomolecules such as protein or chitosan with SeNPs through strategic functionalization could potentially solve these challenges and spawn desired properties from such hybrid, bio-functionalized systems.

Polysaccharides are associated with a variety of physiological functions including immunomodulation, antioxidation, antitumor, anticancer, and anti-cardiovascular disease activities^19^,^20^,^21^,^22^,^23^,^24^. *Dictyophora indusiata*, a saprophytic fungus belongs to the *Phallaceae Corda* family is consumed worldwide. Many active substances from *D. indusiata* (e.g., flavonoids, proteins and polysaccharides) show a range of bioactivities such as antioxidant, anti-tyrosinase, immunoregulation, and anti-inflammatory activities^25^,^26^. A recently extracted, novel polysaccharide DP1 showed strong immunomodulatory activity such as increasing the secretion of interleukin (IL-6), tumor necrosis factor (TNF-α), and nitric oxide (NO) in murine RAW264.7 cells^27^.

Combining the bioactivities, stabilities, and functionalities of SeNPs and DP1 could plausibly generate a peerless hybrid structure with unprecedented properties, bioactivity, specificity, as well as stability for various bio-medicinal applications. In the present study, we demonstrate the synthesis of a novel SeNPs using DP1 as the modifier and stabilizer in a simple redox system with ascorbic acid and sodium selenite. The product was characterized, and the antiproliferative activity against selected human cell lines was examined. The molecular mechanisms involved in the antiproliferative and apoptosis-inducing activities of DP1-SeNPs towards HepG2 cells were also investigated. Compared to other reported functionalization SeNPs, novel DP1 functionalized SeNPs with its unparalleled bio-friendly properties is envisioned to contribute a step-change in futuristic anticancer treatment applications.

## Results and Discussion

### Preparation and characterization of DP1-SeNPs

**Figure 1:**
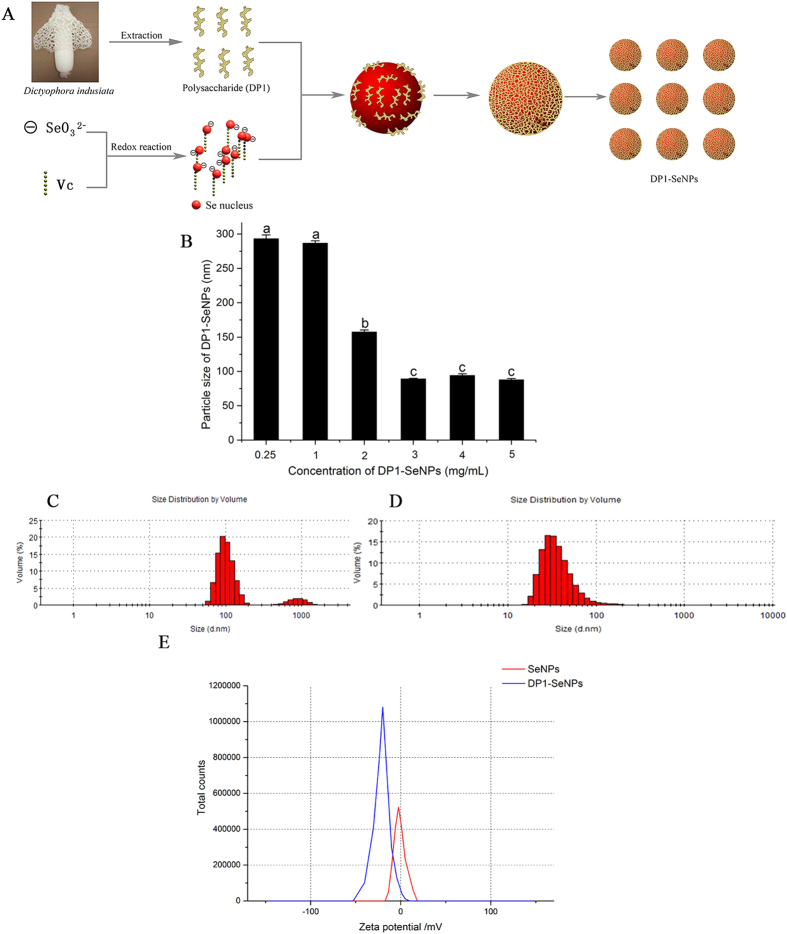
(**A**) Schematic illustration of the preparation of DP1-SeNPs (taken by Wenzhen Liao) (**B**) Size of DP1-SeNPs surface-decorated by different concentrations of DP1, Bars with different characters are statistically different at P < 0.05 level; Size distribution of SeNPs without (**C**) or with (**D**) DP1 (3 mg/mL). (**E**) Zeta potential of SeNPs and DP1-SeNPs.

The synthesis of DP1-SeNPs involved a redox reaction between sodium selenite and ascorbic acid in presence of DP1 modifier, a polysaccharide isolated from *Dictyophora indusiata*. In this redox system, SeO_3_^2−^ was reduced into a Se atom when mixed with ascorbic acid in presence of DP1. The coalescence Se atoms in solution results in the formation of nanoparticles and DP1 molecules, which are chain-like structures with large amounts of hydroxyl groups, can easily conjugate with the surface of the SeNPs to form DP1-SeNPs. The presence of DP1 with large number of hydrophilic moieties on the surface of DP1-SeNPs, increases the stability (due to the negative electrostatic repulsion) and make them easily dispersible in water compared bare SeNPs (Fig. 1A). Bare SeNPs without any stabilizing DP1, easily aggregated and precipitated. The effect of DP1 concentration on the particle size distribution was also studied. DP1 concentration in the ensemble was varied from 0.25 to 5 mg/mL (0.25, 1.0, 2.0, 3.0, 4.0 and 5.0 mg/mL) and Fig. 1B illustrate the variation in the diameter with increasing DP1 content. When the DP1 concentration was less than 1.0 mg/mL, the average diameter of DP1-SeNPs was greater than 250 nm. With the concentration of DP1 increased, the average diameter of Se nanoparticles decreased notably. It was observed that the average diameter of DP1-SeNPs significantly decreased when DP1 went from 1.0 to 3.0 mg/mL. At 3.0 mg/mL, the average diameter of DP1-SeNPs reached ~ 89 nm. No significant change of particle size was observed by further increase in DP1 concentration (from 3.0 to 5.0 mg/mL). These results indicated that 3.0 mg/mL of DP1 was totally chelated with Se nanoparticles, the lower DP1 concentrations (0.25, 1.0, 2.0) were inadequate while higher concentrations (4.0 and 5.0 mg/mL) were excessive for completely surface decoration of Se nanoparticles in this complex reaction system. Thus, 3.0 mg/mL was fixed as the optimal concentration of DP1 in the synthesis.

Figure 1C,D demonstrate the size distribution of SeNPs with or without 3.0 mg/mL DP1. In the absence of DP1, SeNPs showed three main peaks centered around 100, 1000 and 10000 nm (Fig. 1C), while due to the encapsulation activity of DP1, DP1-SeNPs exhibited comparatively smaller dimensions and consequently narrow size distribution curve with an average diameter of 89.4 nm (Fig. 1D).

The zeta potential, also known as the electrokinetic potential, key indicator of stability of nanosystems was employed to evaluate the stability of DP1-SeNPs. Zeta potential measures the repulsive and attractive forces that the particles experience as they approach each other in solution and gave critical information regarding the stability of a nanocomposite. As shown in Fig. 1E, the zeta potential for bare SeNPs was 8.1 mV and it increased to 33.0 mV in presence of DP1 (3.0 mg/mL). The higher the zeta potential of DP1-SeNPs indicate the greater the stability of DP1 functionalized SeNPs. DP1-SeNPs was highly water-dispersible for over 30 days without any aggregation and precipitation observed. The high stability of DP1-SeNPs under physiological condition enabled it to be a candidate for medical applications.

**Figure 2:**
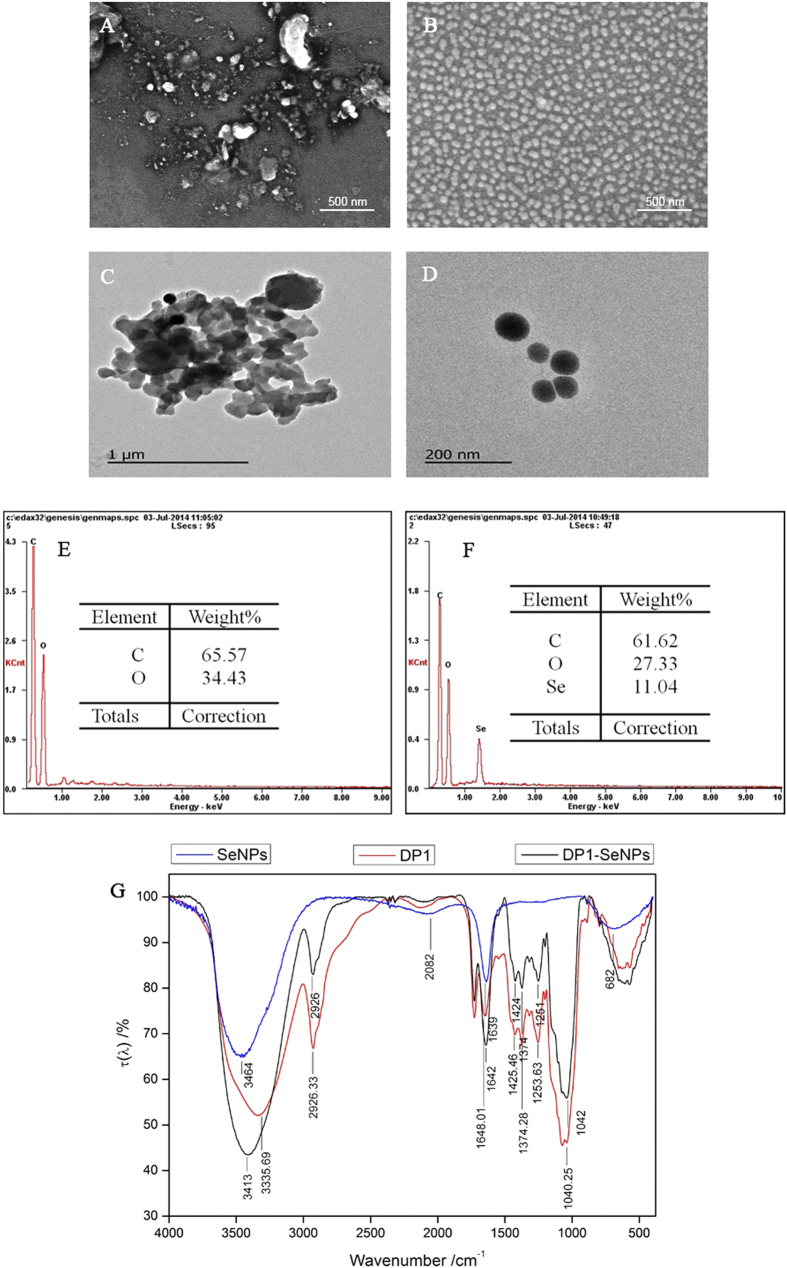
SEM images of SeNPs without (**A**) and with (**B**) DP1; TEM images of SeNPs without (**C**) and with (**D**) DP1; EDX analysis of DP1 (**E**) and DP1-SeNPs (**F**). FTIR analysis of DP1, SeNPs and DP1-SeNPs (**G**).

The morphology of DP1-SeNPs and SeNPs were analyzed using the SEM and TEM. As shown in Fig. 2A,C, SeNPs were highly heterogeneous and aggregated. This is understandable since in the absence of a capping agent, uncontrollable growth, aggregation, and consequent precipitation of metastable nanoparticles is expected. It should be noted that this is just a representative image and a very small fraction of smaller particles (as suggested by DLS) was also observed. However, due to the heavy aggregation and precipitation, the majority of the particles observed under the microscopic were macroscopic in nature. However, due to the intimate capping ability of DP1, the DP1-SeNPs were homogeneous spherical particles (Fig. 2B,D) with relatively smaller diameter.

The surface elemental compositions of DP1-SeNPs were studied using SEM-EDX (Fig. 2E,F). The DP1 mainly contains two elements, C atoms (65.6%) and O atoms (34.4%). The DP1-SeNPs contained Se atoms (11.0%) together with C (61.6%), and O (27.3%) establishing a Se-DP1 conjugation rate of 11.0%.

The DP1-SeNPs was further characterized using FT-IR. The DP1 (Fig. 2G) showed prominent peaks at 3335, 1648, and 1253 cm^−1^ that were assigned to the stretching vibrations of hydroxyl groups, C = O groups, and C-O-C groups, respectively. Similar features were observed in DP1-SeNPs spectrum as well. However, the peaks were slightly blue shifted indicating the conjugation between DP1 and SeNPs through Se-O bonds. The FT-IR spectrum of DP1-SeNPS indicated the stretching vibrations of the hydroxyl groups shifted from 3335 to 3413 cm^−1^; the peak related to the C = O group shifted from 1648 to 1642 cm^−1^; and the peak for the C-O-C group shifted from 1253 to 1251 cm^−1^ compared to pristine DP1.

### Antiproliferative Activity of DP1-SeNPs

**Figure 3:**
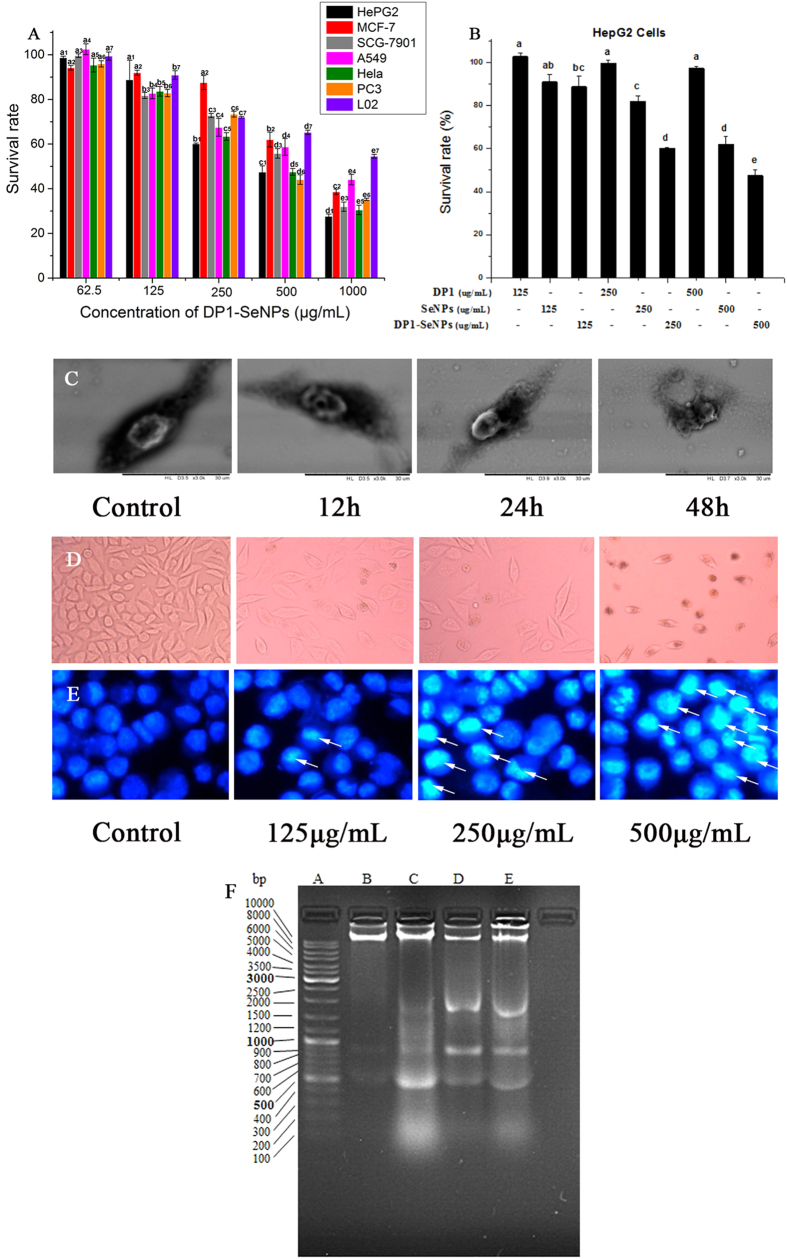
Antiproliferative effects of DP1-SeNPs on selected human cell lines (A). Cells were treated with different concentrations of DP1–SeNPs for 48 h. Bars with different characters are statistically different at P < 0.05 level; (**B**) Antiproliferative effects of DP1–SeNPs, SeNPs and DP1 on HepG2 cells. Cells were treated with different concentrations of DP1–SeNPs, SeNPs or DP1 for 48 h. (**C**) SEM images of HepG2 cells treated with DP1-SeNPs. Cells were treated with 500 μg/mL of DP1-SeNPs for 12, 24 and 48 h. The control group was treated with PBS. (**D**) POM images of HepG2 cells treated with DP1-SeNPs at different concentrations for 48 h. The control group was treated with PBS. (**E**) Photomicrographs of HepG2 nuclear condensation (indicated by the arrows) induced by DP1-SeNPs. Cells were cultured with different concentrations of DP1-SeNPs (125, 250 and 500 μg/mL) for 48 h and detected using the Hoechst 33258 assay. The control group was treated with PBS. (**F**) DNA fragmentation of HepG2 cells treated with 125, 250 and 500 μg/mL of DP1-SeNPs for 24 h. Lane A: DNA maker, lane B: control group, lane C: 125 μg/mL, lane D: 250 μg/mL, and lane E: 500 μg/mL exposed group.

The antiproliferative activity of DP1-SeNPs was evaluated using the MTT assay. Figure 3A shows the significantly inhibited growth of the selected cancer cells (HepG2, MCF-7, SGC-7901, A549, Hela, PC3, and L02) by DP1-SeNPs. Moreover, considerable cancer-specific cytotoxicity was also demonstrated DP1-SeNPs and DP1-SeNPs were able to specifically induce apoptosis of cancer cells (HepG2) but was relatively safe for normal cells (L02). The survival rate of normal human hepatocytes, L02, was higher compared to the cancer cells (HepG2) after treatment with DP1-SeNPs, especially at the highest concentration (500 mg/mL). Among these 7 cell types tested, HepG2 was the most sensitive and its survival rate decreased to 27.7% with 500 μg/mL of DP1-SeNPs. Therefore, the detailed intracellular antiproliferative mechanisms of DP1-SeNPs was investigated using the HepG2 cell liner.

We further evaluated the antiproliferative effects of DP1-SeNPs, SeNPs, and DP1 against HepG2 cells to investigate the contribution of DP1 surface modification to the antiproliferative activity of SeNPs. As shown in Fig. 3B, SeNPs alone did exhibit certain antiproliferative effect against HepG2 cells, while DP1-SeNPs demonstrated more significantly antiproliferative activity than SeNPs. The main reason for this might be due to the better stability and celluar uptake of DP1-SeNPs than SeNPs. Additionally, no evident antiproliferative effect of HepG2 cells was found by DP1 treatment alone. These results indicated that DP1 surface modification is crucial to enhance the antiproliferative action of SeNPs against HepG2 cells.

The morphological damage of HepG2 cells induced by DP1-SeNPs with different exposure time was examined using time-dependent SEM. Figure 3C illustrates the SEM image of spindle-shaped HepG2 cells not exposed to DP1-SeNPs. However, corresponding HepG2 cells exposed to DP1-SeNPs shrank as incubation time increased and eventually broken apart at 48 h, implying the time-dependent cytotoxicity of DP1-SeNPs.

The morphological changes of the HepG2 cells were examined using the polarized optical microscope as well. The dose-dependent effect of DP1-SeNPs can be observed in Fig. 3D. Again, untreated HepG2 cells demonstrated a spindle shape. As the DP1-SeNPs increased, HepG2 shrunk and changed the shape from spindle to circular, and the number of adhering cells decreased. Large numbers of coke-like apoptotic HepG2 cells were seen after 48 h of treatment with the highest dose (500 μg/mL) of DP1-SeNPs. The results suggested that the DP1-SeNPs could induce dose- and time-dependent morphologic damage of HepG2 cells.

### Induction of Apoptosis by DP1-SeNPs

Apoptosis, also known as programmed cell death, is a genetically-controlled cell death process. Apoptosis is often characterized by certain dominant features including cytoplasm shrinkage, nuclear chromatin condensation, membrane dysfunction, and formation of apoptotic bodies^28^. Previous reports indicated that apoptosis is one of the key mechanisms for the anticancer action of Se-based materials^28^,^29^.

In this study, DP1-SeNPs-induced nuclear changes of HepG2 cells were examined using the Hoechst 33258 assay. Hoechst 33258 is a DNA-specific fluorochrome which emits a blue fluorescence with UV excitation. As shown in Fig. 3D, the nuclei of control HepG2 cells were round with homogeneous blue fluorescence. Whereas, after exposure to DP1-SeNPs, the HepG2 cells shrunk, the nuclear chromatins were condensed, and apoptotic bodies (shown by the white arrow in Fig. 3E) were observed. These results confirmed that DP1-SeNPs-effected cell death of HepG2 is via apoptosis.

DNA fragmentation is another typical biochemical features of apoptosis^30^. The inter-nucleosomal cleavage of DNA induced by DP1-SeNPs was studied using a DNA fragmentation assay. DNA was extracted from HepG2 cells and studied using agarose gel electrophoresis. The electrophoretogram is given in Fig. 3F. While a clear “DNA ladder patterns” of in consecutive DNA fragments were found in DP1-SeNPs treated groups, no “DNA ladder” was found in the control group. In addition, a concentration-dependent increase in DNA cleavage was also observed for the DP1-SeNPs treated samples. These results established that the antiproliferative effect of DP1-SeNPs on HepG2 cells was mainly through the induction of cell apoptosis.

**Figure 4:**
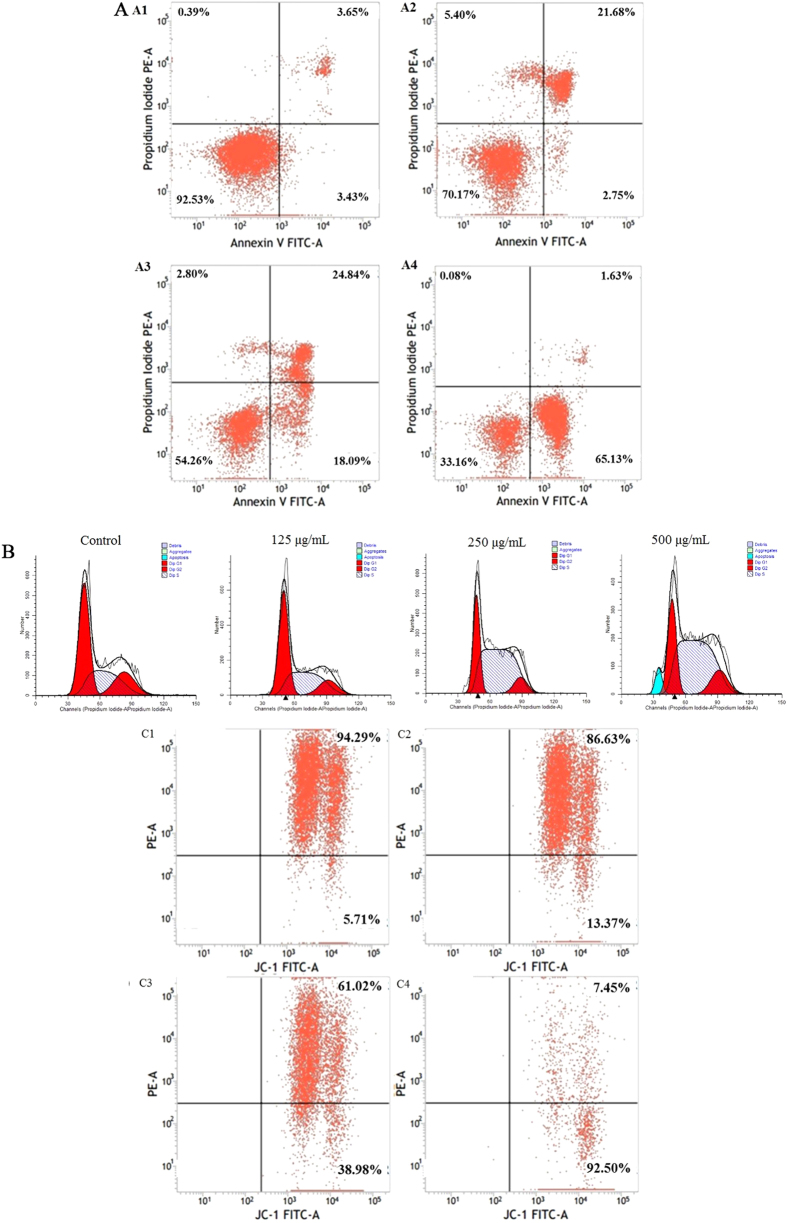
Flow cytometry analysis of HepG2 cells treated with DP1-SeNPs. (**A**) Apoptosis analysis: (A1) Control group; (A2) cells treated with 62.5 μg/mL DP1-SeNPs; (A3) 125 μg/mL; and (A4) 250 μg/mL DP1-SeNPs. The lower left quadrant (LL) represents normal cells, the lower right quadrant (LR) represents early apoptotic cells and the upper left (UL) and upper right (UR) quadrants represent necrosis and late apoptosis cells, respectively. (**B**) Cell cycle distribution of HepG2 cells treated with DP1-SeNPs; (C) Loss of mitochondrial membrane potential of HepG2 cells treated with DP1-SeNPs. (C1) control group; (C2) cells treated with 125 μg/mL; (C3) 250 μg/mL; and (C4) 500 μg/mL DP1-SeNPs. The UR and UL represent the red fluorescence and green fluorescence, respectively.

The apoptosis mode and rates of HepG2 cells was analyzed using flow cytometry (Fig. 4A), and a significant dose-dependent decrease in the proportion of normal cells was found using the Annexin V-FITC/PI staining assay. This test also validated that there is a significant difference between apoptosis of pristine and treated HepG2 cells. While HepG2 cells treated with 125 and 250 μg/mL of DP1-SeNPs had a significant increase in late stage apoptosis as compared with the control samples. Understandably the proportion of early apoptosis increased with 250 and 500 μg/mL of DP1-SeNPs. However, the ratio of necrosis in HepG2 cells showed no distinct changes. These results suggested that DP1-SeNPs has efficient antiproliferative activity both at low- and high- concentrations. While DP1-SeNPs at relative low concentration (250 μg/mL) mainly inhibit HepG2 cell proliferation in late stage apoptosis, the exposure at a higher concentration (500 μg/mL), the inhibition of cell proliferation also occurred in early-stage apoptosis. These results also established that DP1-SeNPs might have the potential to be used as an anti-cancer drug.

### Inducing Cell Cycle Arrest using DP1-SeNPs

Inhibition of cancer cells proliferation with anti-cancer drugs often results in the induction of apoptosis, cell cycle arrest, or a combination of these two modes^31^. To categorically identify the modes of cell antiproliferation by DP1-SeNPs, the PI staining assay using flow cytometry was employed. Figure 4B depicts the content of diploid DNA and their distribution histograms for the HepG2 cells exposed to DP1-SeNPs and Table 1. The data explicitly demonstrated a dose-dependent increase in the percentage of arrested S phase cells, from 32.4% (control) to 64.2% (250 μg/mL). There were no significant changes in the G2/M phase, the percentage of G0/G1 phase arrested cells decreased as the S phase arrested cells increased. Mitosis and cell proliferation were stagnant when cells were arrested in S phase. Thus, it is proved that the antiproliferative activity of the DP1-SeNPs is caused by a combination of apoptosis and S phase cell arrest.

### Mitochondrial Dysfunction Induced by DP1-SeNPs

**Figure 5:**
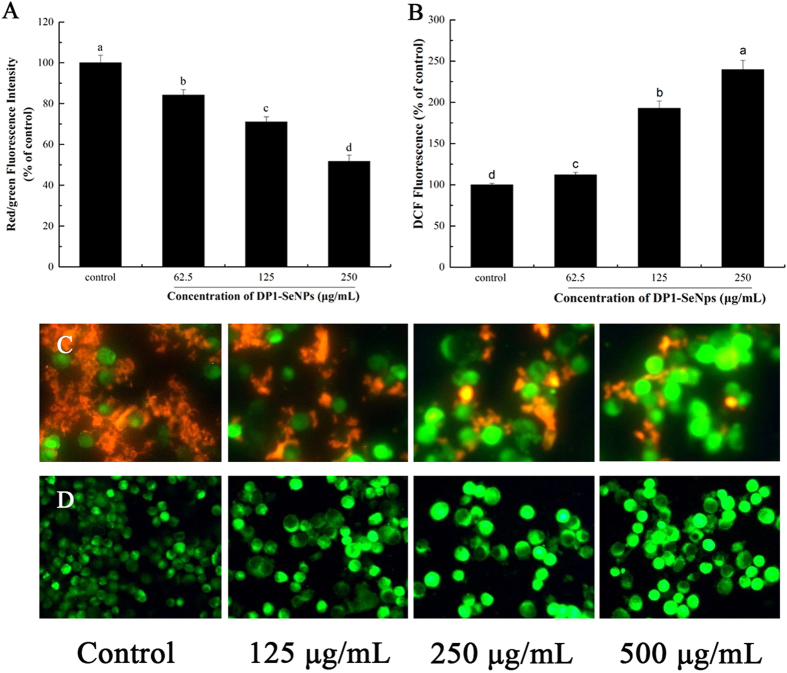
(**A**) Red/green fluorescence intensity with JC-1 staining after treatment with DP1-SeNPs (125, 250 and 500 μg/mL) for 48 h. Bars with different characters are statistically different at P < 0.05 level. (**B**) Intracellular ROS generation induced by DP1-SeNPs after treatment with DP1-SeNPs (125, 250 and 500 μg/mL) for 48 h. Bars with different characters are statistically different at P < 0.05 level; (**C**) JC-1 staining image after 24 h treatment with 125, 250 and 500 μg/mL of DP1-SeNPs. (**D**) Intracellular ROS levels imaged using a fluorescence microscope after treatment with DP1-SeNPs (125, 250 and 500 μg/mL) for 48 h.

Mitochondria participate in apoptotic signalling pathways through the integration of intrinsic and extrinsic apoptotic signals. Loss of the mitochondrial membrane potential through changes in permeability is a step in the induction of apoptosis^32^. Hence, in this study the mitochondrial membrane potential was measured using the JC-1 assay to evaluate the DP1-SeNP induced mitochondrial dysfunction. For this, HepG2 cells treated with different concentrations of DP1-SeNPs were stained with the mitochondria-selective JC-1 dye. In normal cells, JC-1 spontaneously aggregated as J-aggregates at high membrane potential and exhibited red fluorescence, while in the apoptotic cells JC-1 remained as a monomer and manifested green fluorescence due to mitochondrial membrane depolarization^33^. As shown in Fig. 4C, a dose-dependent reduction of the mitochondrial membrane potential was observed using flow cytometry. The red/green fluorescence intensity ratio of the HepG2 cells treated with DP1-SeNPs is shown in Fig. 5A. The ratio decreased with the increase of DP1-SeNPs concentration. This red/green fluorescence intensity was confirmed using a fluorescence microscope. As shown in Fig. 5C, there was a decreased tendency for red fluorescence in DP1-SeNPs treated HepG2 cells compared to the control suggesting that mitochondrial damage is leading to apoptosis in the case of DP1-SeNP treated cells.

### DP1-SeNPs-Induced Mitochondria Dysfunction via ROS Overproduction

Reactive oxygen species (ROS), including the hydroxyl radical (•OH), hydrogen peroxide (H_2_O_2_), singlet oxygen (O_2_), and the superoxide anion radical (O_2_^**−**^•) are mainly generated by the mitochondrial respiratory chain reaction during oxidative metabolism^34^. Overproduction of ROS may result in oxidative damage to the mitochondria, nucleic acids, proteins and lipids, which can eventually cause cell apoptosis^35^,^36^. To evaluate the contribution of ROS overproduction, the intracellular generation of ROS in HepG2 cells treated with DP1-SeNPs was measured using DCFH-DA as a fluorescent probe. DCFH-DA is a fluorescein-labeled dye which can be hydrolyzed by nonspecific intracellular esterases to non-fluorescent DCFH. DCFH is then rapidly oxidized by peroxy radicals into a green fluorescent compound: 2′, 7′-dichlorofluorescein (DCF) which provides a quantitative assay for ROS formation. As shown in Fig. 5D, DP1-SeNP-treated HepG2 cells showed higher, dose-dependent green fluorescence intensity (Fig. 5B) showing that the overproduction of intracellular ROS is a likely cause of mitochondrial degradation.

### DP1-SeNPs Induced Caspase-Dependent Apoptosis

**Figure 6:**
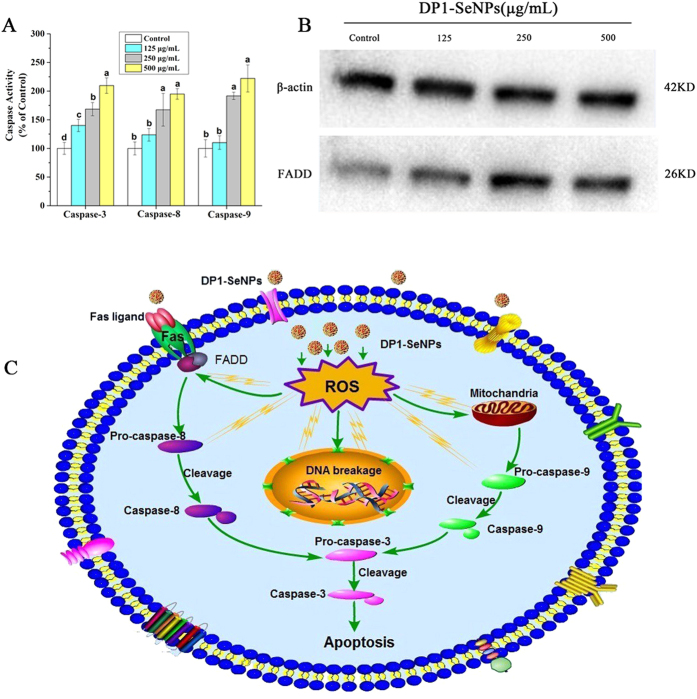
(**A**) Activation of caspase-3, caspase-8 and caspase-9 in HepG2 cells after treatment with DP1-SeNPs (125, 250 and 500 μg/mL) for 48 h. Bars with different characters are statistically different at P < 0.05 level. (**B**) Western blot analysis for the expression of FADD. β-Actin was used as a loading control. The two blots are run under the same experimental conditions. (**C**) The underlying signaling pathway of DP1-SeNPs induced apoptosis in HepG2 cells.

Caspases are involved in cell apoptosis, some as initiators and some as effectors 37. Activation of the caspase cascade can lead to cell apoptosis. Caspase-3 is considered to be the central regulator of apoptosis, while caspase-8 and caspase-9 act as initiators of the death receptor-mediated and mitochondria-mediated apoptotic pathways, respectively^34^,^38^. To determine whether caspase activation is involved in the DP1-SeNPs induced cell death, the activities of the initiator caspases (caspase-8 and caspase-9) and effector caspases-3 were studied using fluorometric assay. Figure 6A shows that exposure of 250 and 500 μg/mL of DP1-SeNPs significantly increased the activities of caspase-3, caspase-8, and caspase-9 in a dose-dependent manner, which suggests that both the death receptor-mediated and mitochondria-mediated apoptotic pathways are involved in the DP1-SeNPs induced apoptosis.

### Activation of Fas-associated Death Domain Protein (FADD) Induced using DP1-SeNPs

FADD is a novel death domain protein which interacts with Fas to initiate apoptosis^39^. FADD, also known as Mort-1, is a signal transducer downstream of Fas^40^. FADD is a component of the tumor necrosis factor receptor type 1 (TNF-R1) and Fas signaling complexes that are involved in TNF-R1-induced and Fas-induced apoptosis^41^. As shown in Fig. 6B, the western blot analysis showed that the expression level of FADD in HepG2 cells was significantly enhanced with increased DP1-SeNPs concentration, indicating that activation of FADD also involved in the DP1-SeNPs induced apoptosis.

## Conclusion

A first-of-its-kind, bio-derived polysaccharide DP1 was employed to encapsulate, stabilize, and enhance the bioactivity of SeNPs via successfully fabricating DP1-SeNPs that showed significant antiproliferative effects on selected human cancer cell lines (HepG2, MCF-7, SGC-7901, A549, Hela, and PC3). Maximum activity was observed against HepG2 cells and hence they were used for the detailed study of the antiproliferative effect SeNPs. It was concluded that atiproliferative activity of DP1-SeNPs is associated with nuclear condensation, DNA cleavage, and S phase cell arrest. It was also established that DP1-SeNPs show a dose-dependent caspases-3, -8, and -9 activation and significantly increased the expression of FADD, implying that the DP1-SeNPs induced apoptotic pathways involves the activation of FADD and caspase-3, -8, and -9 as well. Furthermore, it was proved that DP1-SeNPs can also induce ROS overproduction and mitochondrial dysfunction to generate enhanced apoptosis. Therefore, it was confirmed that the antiproliferative activity of DP1-SeNPs was both via death receptor-mediated and mitochondria-mediated apoptotic pathways. On the basis of these results, it is envisaged that the novel DP1-SeNPs complex may be a promising chemopreventive and chemotherapeutic agent and warrant further study.

## Materials and Methods

### Materials

The fresh fruiting bodies of *D. indusiata* were purchased from Zhijin, Bijie City, Guizhou Province, China. Ascorbic acid, sodium selenite (Na_2_SeO_3_), 5,5′,6,6′-tetraethylimida-carbocyanine iodide (JC-1), 2′,7′-dichlorofluorescein diacetate (DCFH-DA), 4′,6-diamidino-2-phenyindole (DAPI), Diethylpyrocarbonate (DEPC), thiazolyl blue tetrazolium bromide (MTT) and bicinchoninic acid (BCA) kits were purchased from the Sigma Company (St. Louis, MO, USA). The human hepatocarcinoma HepG2 cell line, the human pulmonary carcinoma A549 cell line, the human cervical carcinoma Hela cell line, the human breast carcinoma MCF-7 cell line, and the human prostate carcinoma PC3 cell line were obtained from the American Type Culture Collection (ATCC, Rockville, Md, USA). The human gastric carcinoma SGC-7901 cell line and the human normal liver HL-7702 (L02) cell line were procured from the Medical College of Sun Yat-Sen University (Guangzhou, Guangdong, China). Phosphate-buffered saline (PBS, pH 7.4), streptomycin, penicillin, heat-inactivated fetal bovine serum (FBS), and Dulbecco’s modified Eagle’s medium (DMEM) were purchased from Gibco Life Technologies (Grand Island, NY, USA). Fas-associated death domain protein (FADD) antibody and β-actin were obtained from Cell Signaling Technology (Beverly, MA, USA). Goat anti-rabbit IgG was purchased from Millipore (Millipore, Bedford, MA, USA). The substrates for caspase-3 and 7: Ac-DEVD-AMC, caspase-8: Ac-IETD-AFC and caspase-9: Ac-LEHD-AFC were purchased from Calbiochem (La Jolla, CA, USA). The 6-well and 96-well plates were obtained from Corning Inc. (Corning, NY, USA). Sephadex G-200 and DEAE (diethylaminoethyl)-52 cellulose were purchased from GE Healthcare Life Science (Piscataway, NJ, USA). Ultrapure water was prepared using a Milli-Q water purification system (Millipore). All the other chemical reagents used in the present study were of analytical grade.

### Preparation of Polysaccharides from *Dictyophora indusiata*

The fruit body of *D. indusiata* was dried at 40 °C for 2 h and crushed using a high-speed disintegrator (DFT-50, Lingda Mechanics Co, Zhejiang, China). The powder of *D. indusiata* was mixed with ultrapure water at a ratio of 1:30 (w/v) and exacted for 2 h at 100 °C. The resultant solution was centrifuged at 4,000 × g for 15 min (Sorvall Stratos, Thermo Scientific, Asheville, NC, USA). The supernatant was collected and deproteinated using the Sevag method^25^. This deproteinizing procedure was repeated 10 times. The resulting solution was precipitated with 3 volumes of ethanol at 4 °C and kept overnight. The sediment was collected by centrifugation (4,000 × g, 15 min) and lyophilized (R2L-100KPS, Kyowa Vacuum Engineering, Tokyo, Japan). The crude polysaccharides were further purified using DEAE-52 cellulose column chromatography using ultrapure water as the eluent. Then further separated by Sephadex G-200 column chromatography again eluted using ultrapure water. The final product was the polysaccharides fraction DP1. This was dialyzed against ultrapure water, lyophilized and used within 3 months.

### Preparation of DP1-SeNPs

The lyophilized DP1 was dissolved in ultrapure water to a final concentration of 3 mg/mL. The DP1 was mixed with 1 mmol/L sodium selenite solution (1:1 (v/v)) with magnetic stirring. A 4 mmol/L ascorbic acid solution (1:8 (v/v)) was added with stirring in the dark for 24 h at 45 °C. The final solution was dialyzed with 3 kDa (according to the manufacturer) cutoff dialysis bag (Spectrum Medical Industries, Inc., Los Angeles, CA, USA) against ultrapure water until no unbound Se was detected by ICP-AES analysis^34^. The DP1-SeNPs solution was lyophilized and stored at −20 °C before use within 1 month.

### Characterization of DP1-SeNPs

#### Scanning Electron Microscope (SEM)

The surface morphology of the DP1-SeNPs was observed using a SEM (Model 1530VP, LEO, Oberkochen, Germany). A drop of DP1-SeNPs (250 μg/mL) solution was spread on a glass slide to obtain a thin film. This was sputter-coated with gold and placed in the SEM.

#### Transmission Electron Microscopy (TEM)

DP1-SeNPs solution was prepared by sonic oscillation and spreaded onto a holey carbon film on copper grids. The micrographs were obtained by a TEM (Model JEM-2100F, Bruker, Germany) operated at an accelerating voltage at 80 kV.

#### Energy Dispersive X-ray (EDX)

The elemental composition of DP1-SeNPs was obtained using an energy dispersive EX-250 system (Horiba Ltd, Kyoto, Japan) X-ray spectrometer (EDX; Voyager III, NORAN Instruments, Inc., Middleton, WI). The powder sample of DP1-SeNPs was spread out and then attached to the specimen holder using double-sided adhesive tape and examined by EDX spectrometer.

### Size Distribution and Zeta Potential Analysis

The size distribution and zeta potential of DP1-SeNPs were characterized using a Zetasizer Nano ZS particle analyzer (Malvern Instruments Limited, Malvern, UK). Different concentration of DP1-SeNPs solutions was placed in the analyzer for the measurement of average diameter and zeta potential.

### Fourier Transform Infrared (FT-IR) Assay

The infrared spectra of DP1-SeNPs were characterized using a Fourier transform infrared (FT-IR) spectrophotometer (Bruker, Ettlingen, Germany) in the range of 400–4000 cm^−1^ according to the KBr-disk method^25^.

### Cell Culture

The selected human cell lines including HepG2, MCF-7, SGC-7901, PC3, A549, Hela and L02 were all maintained in DMEM plus with 10% fetal bovine serum (FBS), 100 U/mL of penicillin and 100 μg/mL of streptomycin, and incubated at 37 °C in a 5% CO_2_ humidified atmosphere.

### Antiproliferative Activity Determinations using the MTT assay

The antiproliferative activity of DP1-SeNPs on the selected human cell lines was determined using the MTT assay. Briefly, cells were seeded at a density of 5 × 10^4^ cells/mL in a 96-well culture plate and incubated at 37 °C for 24 h. Different concentrations of DP1-SeNPs, SeNPs, and DP1 were added at 200 μL per well, and incubation continued for 48 h. After incubation, 20 μL of MTT solution (5 mg/mL) was added to each well and incubated for another 4 h at 37 °C. Then the medium containing MTT was removed and replaced with 100 μL of DMSO to dissolve the formazan formed in the surviving cells with intact mitochondria. Finally, the cell plate was placed on a microplate spectrophotometer (Versamax, Molecular Devices, Sunnyvale, CA, USA) and the absorbance was measured at 570 nm. The survival rate of the selected cells was quantified using the following formula:


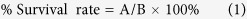


where A and B stand for the absorbances of the treated cells and control cells, respectively.

### Cell Morphology Analysis using the Polarized Optical Microscope (POM)

The morphological changes of HepG2 cells induced by DP1-SeNPs were observed using a POM (Nikon Eclipse LV100 POL (Nikon Corporation, Tokyo, Japan)). Cells were seed at a density 5 × 10^4^ cells/well on 6-well culture plates and incubated at 37 °C for 24 h. Different concentrations (125, 250 and 500 μg/mL) of DP1-SeNPs were added to the cells and incubated for 48 h. Cells without treatment of DP1-SeNPs were used as the control. Then the cell plates were placed on the object stage of the POM and investigated.

### Cell Morphology Analysis using the SEM

The morphological changes of HepG2 cells were further analyzed using the SEM (Hitachi TM3000, Hitachi Ltd., Tokyo, Japan). A sterile coverslip was placed in the bottom of each well of the 6-well plate. HepG2 cells were seed on top of the coverslips at a density of 4 × 10^4^ cells/well and incubated for 24 h. DP1-SeNPs at 250 μg/mL was added to the HepG2 cells for different time interval (12, 36 and 48 h). Then the cells were washed with PBS (pH 7.4) three times and immobilized with 2.5% glutaraldehyde for 2 h. Finally, the cell slides were transferred to the specimen holder of the SEM and observed.

### Nuclear Morphology Analysis using the Hoechst 33258 Assay

HepG2 cells were seeded in 6-well culture plates (5 × 10^4^ cells/well) and treated with various concentrations (125, 250 and 500 μg/mL) of DP1-SeNPs (2 mL) for 48 h. Then the cells were washed with PBS (pH 7.4) three times and tryptic (with 500 μL of trypsin) digested for 3 min at 37 °C and transferred into centrifuge tubes. Then the cells were fixed with 4% methanol at 4 °C for 10 min and collected by centrifugation (2000 g/min, 10 min). The harvested cells were re-suspended with 30 μL of PBS and spread on a glass slide. The slides were air-dried and stained with 100 μL of Hoechst 33258 for 10 min. Slides were then washed with PBS three times and observed using a fluorescence microscope (excitation/emission, 350/460 nm) (Nikon Eclipse 80i, Nikon).

### Apoptosis and Cell Cycle Analysis using Flow Cytometry

The apoptosis of HepG2 cells was examined using an Annexin V-FITC/PI apoptosis detection kit (Bipec Biopharma Corporation, City, MA. USA) and analyzed using a flow cytometry FACS™ Universal Loader (BD Becton Dickinson, Franklin Lakes, NJ, USA). Briefly, HepG2 cells with or without DP1-SeNPs were harvested after tryptic digestion as described above. Then the cells were suspended in the binding buffer provided and divided into two equal portions. One portion was stained using FITC-labeled Annexin V and propidium iodide (PI) and incubated at room temperature in the dark for 15 min. The other part was used as the blank control. The stained cells were analyzed using flow cytometry at an excitation wavelength of 488 nm. The signal from the FITC was read at 516 nm while the signal from the PI was read at 560 nm. Data was analyzed using the Cell Quest Research Software (Becton Dickinson).

The cell cycle distribution of HepG2 cells was detected using a PI staining assay and analyzed using the flow cytometer. Briefly, HepG2 cells with or without DP1-SeNPs were harvested and washed three times with PBS. The treated cells were then fixed using a 70% alcohol solution and kept in the dark at −20 °C for 12 h, then the cells were dyed using the 10 μL PI solution with RNase (100 μg/mL) and kept in the dark at 37 °C for 30 min. Tagged cells after washing with PBS three times were analyzed using the flow cytometer. Ten thousand events were recorded for each sample. Cell cycle distribution of HepG2 was determined by the amount of PI intercalated with DNA and analyzed by the Cell Quest Research Software as above.

### Determination of Mitochondrial Membrane Potential (ΔΨm)

The ΔΨm of HepG2 cells was determined using JC-1 assay and analyzed using flow cytometry, fluorescence microscopy and a Fluoroskan ascent microplate fluorometer (Thermo Electron Corporation, Vantaa, Finland). JC-1 is an ideal fluorescent probe widely used in the determination of ΔΨm. It can accumulate in the actively respiring mitochondria if there is a membrane potential. JC-1 forms ‘J-aggregates’ at a high mitochondrial membrane potential, which shows a different characteristic absorption and emission spectra compared to the JC-1 monomers at a low membrane potential. HepG2 cells were incubated with or without DP1-SeNPs for 48 h in 6-well plates. The cells were harvested by trypsinization and washed three times with PBS (pH 7.4), then the cells were stained using 10 mg/mL JC-1 for 10 min in the dark at 37 °C. The stained cells were washed and suspended in PBS before flow cytometric analysis; excitation/emission, 488/525 (green) and 590 nm (red). The fluorescence intensity of the stained cells was determined using the fluorometer (excitation/emission: 590/525 nm). The JC-1 stained cells were also spread onto a glass slide and observed using a fluorescence microscope with Argon-ion 488 nm laser excitation (Nikon Eclipse 80i).

### Intracellular Reactive Oxygen Species (ROS) Production

The intracellular ROS generation of HepG2 cells was determined using the DCFH-DA assay as described previously^35^. Briefly, HepG2 cells were harvested and washed three times with PBS (pH 7.4). The cells were incubated with 10 μM DCFH-DA at 37 °C for 30 min. Then the cells were washed three times with PBS (pH 7.4) to wash off excess DCFH-DA. Different concentrations (125, 250 and 500 μg/mL) of DP1-SeNPs were added at 37 °C for 30 min. The intracellular ROS level was measured using the fluorometer (excitation/emission, 488/525 nm). The fluorescence intensity of cells was also determined (excitation/emission, 495/525 nm) in the fluorescence microscope.

### DNA Fragmentation Assay

HepG2 cells were treated with different concentrations (125, 250 and 500 μg/mL) of DP1-SeNPs at 37 °C for 48 h. The treated cells were washed three times with PBS (pH 7.5) and lysed with lysis buffer (0.5% Triton X-100, 10 mM EDTA and 10 mM Tris-HCl, pH 7.5) for 25 min. The cell-lysates were incubated at 37 °C for 30 min and then kept at 55 °C for 1 h. After that, the lysates were dissolved in a proteinase K (0.25 mg/mL) and RNase (0.03 mg/mL) solution. The nuclear DNA of the HepG2 cells was then extracted using phenol-chloroform mix and purified with isopropyl alcohol and kept at −20 °C for 20 min. The DNA samples were washed with 70% ethanol three times and resuspended in DEPC treated water. The DNA samples were stained with ethidium bromide (1 mg/mL) and analyzed using agarose gel (2%) electrophoresis. The electrophoretograms were captured by a gel image processing system (Tanon 1600; Tanon, Shanghai, China).

### Caspase Activity Assay

HepG2 cells with or without DP1-SeNPs (125, 250 and 500 μg/mL) were harvested and suspended in lysis buffer and incubated on ice for 1 h. The cell lysates were centrifuged at 4 °C (11,000 × *g*, 30 min). After centrifugation, supernatants were collected, and the protein concentration was measured immediately using a BCA assay kit (in accordance with the manufacturer’s instructions). For the caspase activity assay, the cell lysates were placed in 96-well plates and incubated with the specific caspase substrates (Ac-DEVD-AMC for caspase-3, Ac-IETD-AMC for caspase-8, and Ac-LEHD-AMC for caspase-9) at 37 °C for 1 h. The activity was determined using the fluorimeter (excitation/emission 380/440 nm).

### Western Blot Analysis

HepG2 cells were lysed using the lysis buffer after treatment with or without DP1-SeNPs. The cell-lysates were centrifuged at 4 °C (13,000 × *g*, 20 min) to obtain the total cellular proteins. The protein concentration was determined using the BCA assay kit. Equal amounts of protein were subjected to electrophoresis in 12% tricine gels and then transferred to a nitrocellulose membrane and blocked with 5% non-fat milk in Tris-buffered saline Tween-20 (TBST) buffer for 1 h at room temperature. Then the membranes were incubated with primary antibodies at 1:1000 dilution in the 5% non-fat milk solution overnight at 4 °C with continuous stirring. Then the membranes were incubated with secondary antibodies conjugated with horseradish peroxidase at 1:2,000 dilution for 1 h at room temperature and washed three times with TBST buffer. Protein bands were visualized on X-ray film using an enhanced chemiluminescence detection FADD, and β-actin was used to standardize the protein content of each lane. The chemiluminescence signals were imaged using the ChemiDoc XRS system (Bio-Rad Laboratories, Hercules, CA, USA).

### Statistical Analysis

All experiments were carried out at least in triplicate and results were expressed as mean ( ± standard deviation). Statistical analysis was done using SPSS 19.0 software (IBM Corporation, NY, USA). The differences between the control and the experimental groups were analyzed using the two-tailed Student’s t-test. Significance was determined at P < 0.05 by analysis of variance. The difference between three or more groups was analyzed by one-way ANOVA for multiple comparisons.

## Additional Information

**How to cite this article**: Liao, W. *et al.* Biofunctionalization of Selenium Nanoparticle with *Dictyophora Indusiata* Polysaccharide and Its Antiproliferative Activity through Death-Receptor and Mitochondria-Mediated Apoptotic Pathways. *Sci. Rep.* **5**, 18629; doi: 10.1038/srep18629 (2015).

## Figures and Tables

**Table 1:** Effect of DP1-SeNPs on the HepG2 cells in the cell cycle.

Dip of HepG2 Cells (%)	Control	DP1-Se (μg/mL)		
		125	250	500
G0/G1	47.69	47.49	27.04	24.80
S	32.43	39.08	64.17	62.71
G2/M	19.88	13.43	8.79	12.49
